# Song characteristics track bill morphology along a gradient of urbanization in house finches (*Haemorhous mexicanus*)

**DOI:** 10.1186/s12983-014-0083-8

**Published:** 2014-11-12

**Authors:** Mathieu Giraudeau, Paul M Nolan, Caitlin E Black, Stevan R Earl, Masaru Hasegawa, Kevin J McGraw

**Affiliations:** School of Life Sciences, Arizona State University, Tempe, AZ 85287-4501 USA; Present address: School of Biological Sciences A08, University of Sydney, Sydney, NSW 2006 Australia; Department of Biology, The Citadel, Charleston, SC 29409 USA; Department of Biology, The College of Charleston, Charleston, SC 29424 USA; Global Institute of Sustainability & School of Sustainability, Arizona State University, Tempe, AZ 85287-5402 USA; Graduate School of Life and Environmental Sciences, University of Tsukuba, 1-1-1 Tennoudai, Tsukuba-shi, Ibaraki 305-8572 Japan

**Keywords:** Urban impacts, Bill shape, Singing behavior, Noise pollution, Vocal communication

## Abstract

**Introduction:**

Urbanization can considerably impact animal ecology, evolution, and behavior. Among the new conditions that animals experience in cities is anthropogenic noise, which can limit the sound space available for animals to communicate using acoustic signals. Some urban bird species increase their song frequencies so that they can be heard above low-frequency background city noise. However, the ability to make such song modifications may be constrained by several morphological factors, including bill gape, size, and shape, thereby limiting the degree to which certain species can vocally adapt to urban settings. We examined the relationship between song characteristics and bill morphology in a species (the house finch, *Haemorhous mexicanus*) where both vocal performance and bill size are known to differ between city and rural animals.

**Results:**

We found that bills were longer and narrower in more disturbed, urban areas. We observed an increase in minimum song frequency of urban birds, and we also found that the upper frequency limit of songs decreased in direct relation to bill morphology.

**Conclusions:**

These findings are consistent with the hypothesis that birds with longer beaks and therefore longer vocal tracts sing songs with lower maximum frequencies because longer tubes have lower-frequency resonances. Thus, for the first time, we reveal dual constraints (one biotic, one abiotic) on the song frequency range of urban animals. Urban foraging pressures may additionally interact with the acoustic environment to shape bill traits and vocal performance.

**Electronic supplementary material:**

The online version of this article (doi:10.1186/s12983-014-0083-8) contains supplementary material, which is available to authorized users.

## Introduction

Humans continue to urbanize Earth’s landscape and alter wildlife habitat in many ways [1-5]. Many species suffer from anthropogenic disturbance, leading to depleted urban biodiversity, although some populations thrive and expand in cities [3,6]. Factors such as human activity [7], pollution exposure [8], artificial lighting [9], elevated temperature [10], and food and water availability [11] can directly impact wildlife success in urban areas [12].

Among the unique conditions that animals experience in urban habitats, ambient city noise is key because it can limit the sound space available for animals to communicate using acoustic signals [13,14]. Many animal species, and especially birds, use acoustic signals to attract mates and/or communicate with competitors [15,16]. Low-frequency urban noise may overlap with songs of native species, limiting sound reception and ultimately even decreasing fitness and population viability [17].

Recent studies have shown that some bird species adjust their song characteristics − specifically by increasing their minimum frequency − to be heard in a noisy urban environment [13,18-20]. However, the ability to make such song modifications may be constrained by several morphological factors, including bill gape, size, and shape [21-25]. During sound production, the vocal tract (i.e. trachea, larynx, and bill) acts as a resonance chamber for the sound frequencies produced by the syrinx [26-28], and subtle variation in bill size/shape and vocal tract morphology affects sound production [22]. Generally, birds with longer, deeper, and wider beaks produce songs with significantly lower minimum frequencies, maximum frequencies, and frequency bandwidths [29]. Ultimately, such bill morphological factors may limit the degree to which certain species can vocally adapt to urban settings; however, to our knowledge, the few studies that have investigated covariance between avian bill and song traits [30,31,24] have been done outside of an urbanization context.

Bill shape in birds is also strongly associated with diet (e.g. short and thin in insectivores, deep and hooked in granivores and carnivores; [32]), such that foraging pressures can work either with or against directional selection on bill size/shape for song production [23,24]. Urban environments offer novel foraging opportunities that may shift bill morphology, and in fact city effects on bill morphology have been documented in house finches, *Haemorhous mexicanus* [33], whereby bill size increased in urban birds perhaps as a function of the availability of larger, harder-to-husk seeds at backyard bird feeders. This modification of bill shape/size may strongly impact song characteristics in urban birds. What is now needed is an integrated understanding of the relationship between bill morphology and song output in the context of urbanization.

Here, we examined the relationship between song characteristics and bill morphology in house finches along a gradient of urbanization in the Phoenix (Arizona, USA) metropolitan area. Song is a sexually selected trait in this species [34], with females preferring to mate with males that sing more, longer songs. For the first time in studies examining urban impacts on animal signals, we quantified a series of different metrics of urbanization, including human population density and seven measurements describing land-use patterns within the 1-km radius around each of our trapping sites [35], to assess how these factors may be associated with bill shape/size and song characteristics. Based on previous studies, we predicted that minimum song frequency would be associated with urban background noise [13,18,19] and that bill size (length, width, and height) would increase in urban habitats [32]. In addition, given that the angle between the upper and lower mandibles should decrease with an increase in bill length (considering a similar aperture at the bill tip), urban birds with longer bills should have a proportionally longer orotracheal cavity with a reduced high resonating frequency compared to rural individuals with shorter bills [22]. Thus, the shift in bill morphology in urban birds should be associated with a decrease in the highest song frequency produced in urban compared to desert areas. To summarize, we predicted a reduction in the song frequency range for finches in human-modified areas, due to both an increase in minimum frequency in response to the urban background noise and a decrease of the highest song frequency associated with the longer bills of urban birds.

## Results

### Habitat description

Using principal component analysis (PCA), urbanization scores were generated using the data for the 8 variables cited below (7 land use variables and human population density). PCA indicated that three PCs captured >84% of habitat variation. PC1 summarized 47% of the variance, while PC2 and PC3 summarized 24% and 13% of the variance, respectively. PC1 correlated negatively and strongly (component loading >94%) with the percentage of land covered by native undisturbed (desert) habitat. PC2 correlated positively and strongly (component loading >81%) with the percentage of land covered by cultivated vegetation. Finally, PC3 was positively correlated with the percentage of land covered by native vegetation (component loading >74%, Table 1).

**Table 1:** **Characteristics of the sites at which we studied house finches in Maricopa County, USA**

**Capture site**	**Phoenix downtown**	**ASU campus**	**Mesa organic farm**	**Crossroads district park**	**Chandler neighborhood**	**Phoenix zoo**	**South mountain park**	**Estrella mountain regional park**
**City**	Phoenix	Tempe	Mesa	Gilbert	Chandler	Phoenix	Phoenix	Goodyear
**Geographical coordinates**	33°27’N 112°03’W	33°25’N 111°55’W	33° 27’N 111° 49’W	33° 19’N 111°43’ W	33° 18’N 111°55’ W	33°27’N 111°57’W	33°21’N 112°4’W	33° 25’N 112°25’ W
**Number of humans living within 1 km of the study site**	7291	10385	4600	17175	3948	50	1001	11
**Sample size bill measurements**	23	20	21	23	22	21	22	20
**Sample size for song analyses**	9	10	-	11	10	13		10
**Mean song frequencies: lowest, highest and range in Hz (SE)**	2162 (47), 6573 (206), 4411 (234)	2137 (55), 6446 (137), 4310 (188)	-	1844 (43), 5890 (195), 4045 (199)	2008 (60), 6212 (182), 4205 (202)	1968 (24), 6720 (112), 4752 (125)	-	1858 (60), 6806 (182), 4949 (200)
**Mean bill size: Length, height, and width in mm (SE)**	9.84 (0,076), 8.16 (0.043), 7.26 (0.041)	9.85 (0,062), 8.00 (0.037), 7.17 (0.040)	9.71 (0.072), 8.12 (0.044), 7.22 (0.047)	10.04 (0.072), 8.06 (0,059), 7.09 (0.055)	10.05 (0,083), 8.04 (0.042), 7.07 (0.041)	9.71 (0.056), 8.17 (0.050), 7.28 (0.042)	9.76 (0.057), 8.14 (0.046), 7.28 (0.070)	9.44 (0.073), 8.14 (0.047), 7.29 (0.043)
***Habitats (% of land covered by):***								
**Cultivated vegetation and grass**	0.00	0.00	3.44	9.07	0.67	8.05	0.05	1.11
**Disturbed (Mesic and Xeric vegetation)**	57.55	38.79	48.72	40.28	59.53	9.01	17.26	1.73
**Compacted soil (prior agriculture or not)**	0.78	1.01	4.5	1.2	5.47	1.99	1.81	0.67
**Disturbed (commercial, industrial, asphalt)**	30.11	48.38	22.51	21.31	20.82	18.64	7.85	2.87
**Undisturbed**	10.55	9.56	14.45	12.05	10	57.45	68.89	67.44
**Vegetation**	1.01	2.25	6.37	3.32	3.47	3.16	4.12	3.62
**River gravel and water**	0	0	0	3.97	0.04	1.68	0.03	22.55

### Urbanization and morphometrics

Tarsus length and body mass were not correlated with bill morphometrics (all P > 0.39). However, tarsus length (rho = 0.76, P = 0.03) was correlated with urbanization PC2 scores, such that birds captured from sites where more land was covered by cultivated vegetation had longer tarsi.

**Figure 1:**
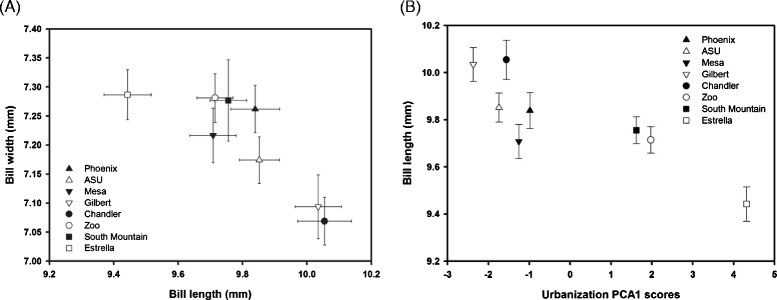
**Relationship between (A) the average bill width and length at each of our eight study sites for which we gathered data on bill traits (±SE) and (B) the average bill length and the urbanization PC1 scores at each of our eight study sites for which we gathered data on bill traits (±SE).**

Bill length (rho = 0.76, P = 0.03; Figure 1) was positively correlated with urbanization PC1 scores, while bill width (rho = −0.79, P = 0.02) was negatively correlated with this urbanization metric (PCA 1 vs bill height: rho = −0.52, P = 0.18). Thus, bill length increased and bill width decreased at sites where less land was covered by native undisturbed habitat. None of the bill traits were correlated with urbanization PC2 and PC3 scores (all P > 0.49).

Using site averages, we found a significant negative relationship between bill width and length, (rho = −0.86, P = 0.006; Figure 1) and a positive association between bill width and height (rho = 0.76, P = 0.03). Bill height was not significantly linked to bill length at the population level (rho = −0.55, P = 0.16).

### Urbanization and song characteristics

Mean maximum frequency of background noise at each site was negatively correlated with urbanization PC2 scores (rho = −0.83, P = 0.04) but not with urbanization PC1 (rho = 0.03, P = 0.96) or PC3 scores (rho = 0.60, P = 0.21). Thus, background noise frequency was higher at sites with less land covered by vegetation.

**Figure 2:**
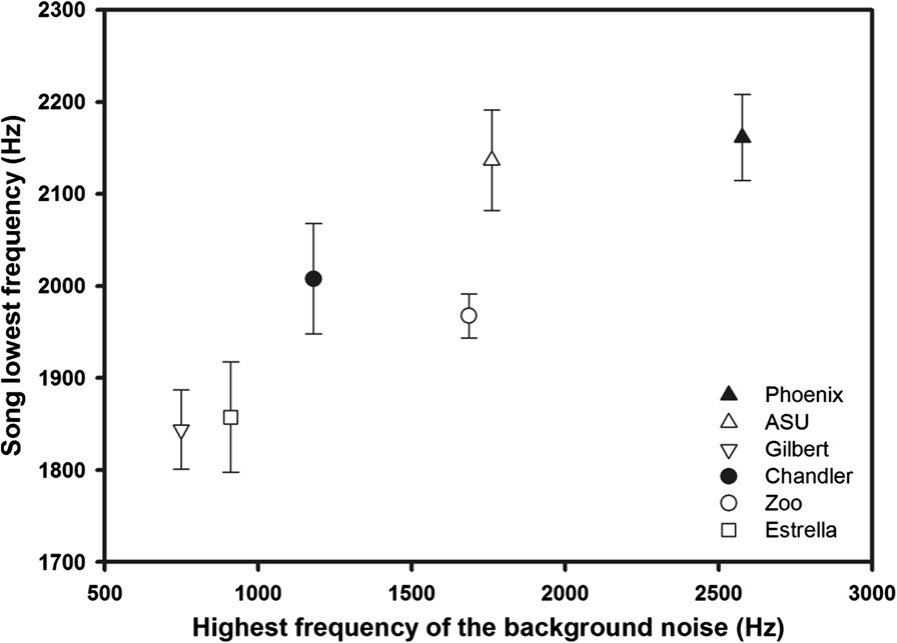
**Relationship between the average song lowest frequency and the highest frequency of the background noise at the six study sites for which we gathered song data (±SE).**

Using site averages, minimum song frequency was significantly positively correlated with maximum frequency of background noise (rho = 0.94, P = 0.005; Figure 2). None of the other song characteristics were related to maximum background-noise frequency (all P > 0.6). Minimum song frequency was negatively correlated with urbanization PC2 scores (rho = −0.94, P = 0.005), while maximum song frequency (rho = −0.83, P = 0.04) and frequency range (rho = −0.84, P = 0.04) were positively correlated with urbanization PC1 scores. In other words, minimum song frequency increased at sites with less land is covered by vegetation, and maximum frequency and the frequency range decreased at sites with a greater percentage of disturbed habitat.

### Intersite covariance between bill shape and song characteristics

**Figure 3:**
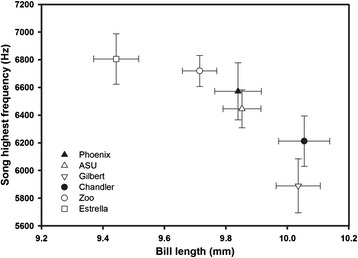
**Relationship between the song highest frequency and the average bill length at the six study sites for which we gathered data on both song and bill traits (±SE).**

**Figure 4:**
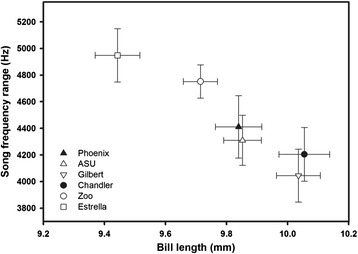
**Relationship between the song frequency range and the average bill length at the six study sites for which we gathered data on both song and bill traits.**

Using site averages, bill length and width, but not height (all P > 0.5), were significantly related to song characteristics; finches sang at lower maximum frequencies (rho = −0.94, P = 0.005; Figure 3) and with a decreased frequency range (rho = −0.94, P = 0.005; Figure 4) at sites where bills were longer and narrower. The lowest song frequencies used by birds were not linked with bill morphology (all P > 0.8). In other words, modifications of bill shape associated with the life in the city were correlated with song maximum frequency and frequency range.

Correlations among bill traits and among song traits within birds and sites are provided in Additional file 1.

## Discussion

We examined relationships between song characteristics and bill morphology in house finches along a gradient of urbanization. We found a gradual increase in bill length and decrease in bill width at progressively more disturbed urban areas. Urban and rural finches differ considerably in foraging and food consumption, given the prevalence of bird seed feeders with large seeds (e.g. sunflower, millet) in suburban and urban areas [36-38,6]. Seeds provided at feeders are argued to require greater bite force to open than natural small seeds of cacti and grasses [33], but this cannot explain why city birds in Phoenix had thinner, longer beaks than did rural finches. The most plausible explanation is that birds with longer beaks gain an advantage in handling large seeds at feeders, while shorter beaks are better suited for processing smaller native seeds. In accordance with this hypothesis, Soobramoney and Perrin [39] showed that passerine species with the smallest bills husked the smallest seeds fastest, while species with the largest bills husked the largest seeds fastest.

More proximately, a modification of bill morphology arises through ontogenetic changes in bill tissue proliferation and migration, a process largely regulated by the expression of bone morphogenetic proteins (BMPs) during early development [40-43]. If juvenile birds in more urban areas are exposed to seeds with different characteristics from those in more rural regions, then different levels of mechanical stress during foraging in early life could drive patterns of BMP production and bill growth (up to 2.5 months after hatching in house finches, [44]). Recently, Badyaev et al. [33] showed that the difference of BMP expression between urban and desert house finches may arise even before hatching. Thus, natural selection may even have favored pre-hatching over-expression of BMP proteins in birds from urban areas (with longer, thinner beaks) compared to natural areas.

We also found that differences in bill morphology along the urban gradient were associated with modifications of song characteristics. The increased bill length and decreased bill width observed in finches from more human-disturbed environments were associated with a decrease in maximum song frequency. These results are in accordance with prior studies showing that larger bills increase the length of the vocal tract, making it more suitable for the production of lower-frequency songs [22,45,29]. However, we did not find a reduction in the minimum song frequency associated with the bill-length increase in urban areas. Conversely, and in accordance with previous studies in house finches and other species, we found a significant positive correlation between the highest frequencies of the background noise and the lowest frequencies of bird song [13,18,19]. Thus, it is likely that urban birds increased their minimum song frequency in order to be heard in a noisy environment, although, with their longer bill, they are probably able to produce lower minimum frequency song than desert birds. Taken together, these results show that the range of song frequencies used by urban birds was drastically reduced (by ca. 20%) compared to those used by rural birds.

In addition to being shaped by static bill morphology, these song patterns may also be due to individual plasticity; house finches have been shown to quickly increase or decrease their minimum song frequencies in response to different experimental noise treatments [46]. However, we are not aware of any studies showing plasticity in the maximum frequencies sung in response to urban noise. Future experiments should examine the role of genetics, development, learning, and vocal-tract plasticity in generating diverse vocal characteristics in an anthropogenic environment.

So what might be the ecological and evolutionary consequences of these song modifications in urban and suburban bird populations? Song traits are key indicators of male quality in many bird species [47]. In the house finch, females showed significant mate preferences based on song characteristics [34]. Therefore, reduction in frequency bandwidth of the signal in response to human activity could have profound reproductive consequences for males. Alternatively, plasticity in female choice could allow receivers to use alternative vocal components that more reliably reflect male quality in the novel environment [48,49]. In line with this hypothesis, Halfwerk et al. (2011, [49]) recently experimentally demonstrated a signaling advantage in male great tits (*Parus major*) for high-frequency songs in noisy conditions, whereas low-frequency songs are likely to be preferred in natural, less noisy environments.

## Conclusions

We have shown for the first time a gradual modification of bill morphology and song characteristics along an urban gradient in populations of house finches. These findings demonstrate the extent to which human activities may strongly impact both the morphology of animals and the associated quality of their sexual signals.

## Methods

### Bill morphology

We used basket traps and Potter traps baited with sunflower seeds to capture 172 adult male house finches at eight sites (two urban, one city park, two suburban, two desert, and one rural; Table 1) in the Phoenix metropolitan area in August-September 2011. At capture, each bird was leg banded with a numbered United States Geological Survey metal ring for individual identification. We also measured body mass (to the nearest 0.1 g with a digital scale), tarsus length (to the nearest 0.1 mm with digital calipers), and bill morphology (to the nearest 0.01 mm with calipers; *sensu* [33]). Bill length was measured from the anterior end of the nostril to the tip of the upper mandible, bill width was measured at the anterior end of the nostril, and bill height was measured in a vertical plane at the anterior end of the nostrils over both mandibles.

### Song measurement and analyses

We recorded songs from adult males during the breeding season (May 2011) from six of the aforementioned study sites (Table 1). We used a Marantz PMD 661 digital recording device (sampling rate: 44,000 Hz; Mahwah, NJ, USA) and a Sennheiser ME 60 directional microphone (Old Lyme, CT, USA) to record the songs. We recorded birds from 0600–1400 hrs. At each location, we listened opportunistically for males to sing, and then recorded them. No playback was used to elicit vocalizations. We approached the birds as closely as possible without disrupting them, and we separated each of our recordings by ≥100 meters to minimize the chance that we inadvertently recorded the same male twice.

Audio recordings were analyzed using Raven Pro 1.4 audio editing software (Cornell University, Ithaca, NY, USA). A song was defined as a set of ≥4 elements with ≥1 second between songs. We isolated 1,165 songs from a total of 79 individuals along the gradient. Using Raven Pro, we generated spectrograms using standardized parameters (Hann window, size =512 samples; DFT size =1024 samples; values below −120 dB were clipped). Recordings of individuals with < 6 songs were discarded (Badyaev et al. [33]). From each song, we extracted the following variables: 1) frequency range, 2) upper frequency and 3) lower frequency. Characteristics were averaged for each bird and then for each study site, to determine the relationship among song characteristics within birds, within sites, and among sites in relation to urbanization (see below) and bill morphology. Once the songs were isolated, we also measured the highest frequency of the background noise during each recording, at a standardized contrast level on the computer display, and calculated the average background noise at each site.

### Habitat description

Most studies on urbanization and animal behavior limit the study sampling to single urban *vs* rural sites (but see [50,51]). However, a more ecologically appropriate sampling scheme is to measure traits at more than one site per habitat type. Moreover, these “urban” and “rural” sites typically vary in many anthropogenic parameters, so it is appropriate to specifically quantify types and degrees of human impact. To assess relationships between finch traits and anthropogenic environmental characteristics, we obtained several urbanization parameters around our eight trapping sites from a local database that is part of the Central Arizona-Phoenix Long-Term Ecological Research program [52-54]: (1) human population density within a radius of 1 km around each trapping site, estimated from the 2010 US Census data; (2) landuse and landcover (LULC, in 2007) variables within the same 1 km radius. From satellite images, we determined percentage of land dedicated to 7 land uses: cultivated vegetation and cultivated grass, river gravel and water, vegetation, disturbed-commercial/industrial and asphalt, undisturbed, disturbed-compacted soil, disturbed-mesic and xeric vegetation residential (see [52-54] for a full description of the LULC types). Using principal component analysis (PCA), urbanization scores were generated using the data for the 8 variables cited above (7 land use variables and human population density, see Results section).

### Statistics

All statistical analyses were carried out with Statistica software (StatSoft, Inc. Tulsa, USA) with α set at 0.05. We ran non-parametric Spearman rank correlations between the three urbanization metrics extracted from the PCA and the average values for bill morphology and song characteristics. We also ran non-parametric correlations among the song traits and among the bill traits within birds and for all birds within a site. As recommended by Nakagawa (2004, [55]), we did not use Bonferroni or similar adjustments to correct for multiple comparisons in order to avoid a reduction of power and an increase of Type II error to unacceptable levels. We did not include the date or time of day in our analyses since song was recorded at every site within a one-week time period and always from 0600–1400 hrs.

## Additional file

Additional file 1: **Supplementary material.**
