# Supplementary material for: Song characteristics track bill morphology along a gradient of urbanization in house finches (Haemorhous mexicanus)
Source: Front Zool. 2014 Nov 12;11:83. doi: 10.1186/s12983-014-0083-8 (PMC4243808; doi:10.1186/s12983-014-0083-8)
Supplement: Supplementary file 1 — Supplementary material. [file 12983_2014_83_MOESM1_ESM.pdf]

SUPPLEMENTARY MATERIAL

Covariation among bill traits within birds and sites

When we considered all birds in our study, we found that bill length and width were not significantly correlated ($F_{1,170}$=0.48, P=0.49), but bill height was significantly positively correlated with both bill length ($F_{1,170}$=5.91, P=0.02) and width ($F_{1,170}$=6.60, P=0.03; table 1).

For the ASU campus and downtown Phoenix sites, only bill length and bill height were positively correlated (*Campus*: rho=2.95, P=0.008; width *vs* length: rho=0.87, P=0.40, height *vs* width: rho=0.95, P=0.35; *Phoenix*: rho=2.14, P=0.04; width *vs* length: rho=1.16, P=0.26, height *vs* width: rho=1.21, P=0.24). In Gilbert, South Mountain, and the Phoenix Zoo, only bill height and width were significantly and positively correlated (*Gilbert*: rho= 2.10, P=0.05; length vs height: rho=0.58, P=0.57; length vs width: rho= 0.54, P=0.59; *South Mountain*: rho= 2.70, P=0.01; length vs height: rho=0.05, P=0.96; length vs width: rho= -0.05, P=0.96; *Zoo*: rho= 0.19, P=0.85; length vs height: rho=0.48, P=0.64; length vs width: rho= 2.18, P=0.04). Finally, none of the bill metrics were significantly correlated in Chandler, Estrella Mountain, and Mesa (*Chandler*: length *vs* height: rho=1.64, P=0.12; length vs width: rho=-0.18, P=0.86; height *vs* width: rho=0.07, P=0.95; *Estrella*: length *vs* height: rho=0.18, P=0.86; length vs width: rho=-0.16, P=0.87; height *vs* width: rho=1.00, P=0.33; *Mesa*: length *vs* height: rho=0.99, P=0.33; length vs width: rho=0.33, P=0.74; height *vs* width: rho=1.40, P=0.18).

Correlation among song traits within birds and sites

When we considered all birds in our study, we found that at our ASU campus site, the three song characteristics were significantly correlated (low freq. vs high freq.: rho= -2.47, P=0.04, low freq. vs freq. range: rho=-4.41, P=0.002, high freq. vs freq. range: rho=7.72, P=10^{-5}). In Phoenix, the frequency range was correlated with the low frequency (rho=-2.48,

P=0.04) and with the high frequency (rho=14.31, $P=10^{-5}$; low freq. vs High freq.: rho=-1.82, P=0.11). In Chandler, Estrella, Gilbert and the zoo, only the high frequency and the frequency range were positively correlated (*Chandler*: rho=4.91, P=0.001, low freq. vs high freq: rho=-0.05, P=0.96; low freq. vs freq. range: rho=-1.30, P=0.23; *Estrella*: rho=7.75, $P=10^{-5}$, low freq. vs high freq: rho=-0.26, P=0.80; low freq. vs freq. range: rho=-1.17, P=0.28; *Gilbert*: rho=12.61, $P=10^{-5}$, low freq. vs high freq: rho=-0.40, P=0.70; low freq. vs freq. range: rho=-0.65, P=0.53; *Zoo*: rho=18.00, $P=10^{-6}$, low freq. vs high freq: rho=-1.17, P=0.18; low freq. vs freq. range: rho=-1.49, P=0.17).
